# Fluctuations and Changes in Acute Phase Reactive Proteins in Fasting and Nonfasting States

**DOI:** 10.1002/jcla.70052

**Published:** 2025-05-10

**Authors:** Ben Huang, Shuxian Miao, Yan Xu, Si‐Jie Qiu, Rui‐Xia Yang, Hua‐Guo Xu

**Affiliations:** ^1^ Department of Laboratory Medicine The First Affiliated Hospital of Nanjing Medical University Nanjing Jiangsu China; ^2^ Branch of National Clinical Research Center for Laboratory Medicine Nanjing Jiangsu China

**Keywords:** acute‐phase reactive proteins, fasting, glucose, nonfasting, OGTT

## Abstract

**Background:**

In clinical practice, acute‐phase reactive proteins (APRPs) are frequently measured at random times. However, it is unclear whether the use of fasting or nonfasting samples affects results. This study aims to investigate the variations of APRPs between fasting and nonfasting conditions.

**Methods:**

This study was conducted based on the oral glucose tolerance test (OGTT) experiment due to standard energy intake and strict time flow. Fifty subjects were enrolled and underwent a 12‐h fasting period before the experiment. Blood samples were collected the following day at baseline (fasting, T0) and 30 (T1), 60 (T2), 120 (T3), 180 (T4) minutes postglucose intake. A total of 250 blood samples were obtained. To quantify clinical fluctuations, percentage bias was calculated, and Bland–Altman plots were employed.

**Results:**

Our observational study demonstrated significant postprandial variations for APRPs. For CRP, 17 (34%) of 50 subjects at T1, 21 (42%) at T2, 23 (46%) at T3, and 16 (32%) at T4 exhibited levels exceeding the maximum allowable error in medical laboratory testing, indicating clinically unacceptable bias. For IL‐6, thirty subjects (60%) at T1, 27 (54%) at T2, 28 (56%) at T3, and 32 (64%) at T4 displayed clinically unacceptable fluctuations. Among other APRPs, the maximum number of subjects exceeding acceptable bias thresholds was 28% (14/50) for procalcitonin, 38% (19/50) for transferrin, 34% (17/50) for prealbumin, and 24% (12/50) for ceruloplasmin.

**Conclusion:**

Clinical fluctuations were observed in the levels of APRPs between fasting and nonfasting states. Clinicians should pay attention to the effects of dietary factors on test results.

## Graphical abstract

This study investigated the clinical changes in acute‐phase reactive protein (APRPs) assays under fasting and nonfasting conditions. The findings revealed significant fluctuations in APRPs levels at different intervals following a standardized diet. Specifically, clinical bias for serum C‐reactive protein (CRP), interleukin‐6 (IL‐6), procalcitonin (PCT), transferrin (TRF), prealbumin (PA), and ceruloplasmin (CER) could exceed maximum allowable error thresholds established by medical laboratories compared with the fasting levels. Consequently, clinicians should be cognizant of dietary influences on test results.

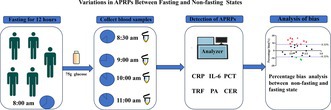

## 1 Introduction

Acute‐phase reaction (APR) refers to the nonspecific complex reaction that occurs shortly after tissue injury caused by infection, trauma, neoplasia, inflammation, and stress [1]. During this process, the inflammatory cells secrete cytokines into the peripheral blood, stimulating the liver cells to produce proteins to participate in the body defense. The proteins are known as the APRPs, and their plasma levels could be rapidly produced and regulated during episodes of inflammation [2, 3]. Clinically, serum APRPs are extensively studied in the context of inflammation and infection. For some hospitalized patients, testing for CRP and other APRPs is often performed during morning blood sampling while the patient is fasting. In some cases, especially in the emergency department, these APRPs testing hardly requires patients to be in a fasting state. However, little is known about the difference and fluctuations of APRPs between fasting and nonfasting serum samples.

According to Pasic et al. [4], fasting could affect 22 types of biochemical markers, including plasma lipoprotein, total protein (TP), albumin (ALB), aspartate aminotransferase (ALT), alanine aminotransferase (AST), uric acid (UA), microelements, and so on [4, 5]. It is worth noting that researchers found clinically significant variations in CRP levels 4 h after the diet [5]. Moreover, a study conducted by Wirth et al. [6] had shown that each 1‐h increase in the duration of the overnight fasting was associated with markedly increased insulin and CRP levels, which suggested that the concentration of APRPs was related to fasting and even fasting time.

Evidence has shown that intermittent fasting (IF) and energy‐restricted diets (ERDs) can reduce systemic inflammatory states [7]. IF is a type of diet that improves metabolism and comes in many forms, including alternate‐day fasting, time‐restricted eating, and other approaches [8]. According to Razavi et al. [9], the alternate‐day fasting diet could be a beneficial approach for the management of body weight, high sensitivity C‐reactive protein (hs‐CRP), and coagulation factors levels in patients with metabolic syndrome. From a biological perspective, intermittent fasting can elicit adaptive cellular responses within and between organs that reduce inflammation, improve glucose regulation, and enhance resistance to oxidative and metabolic stress [10]. A study conducted by Wang et al. [11] has suggested that IF and ERDs could reduce CRP concentrations, especially in overweight and obese individuals. However, Hassane et al. [12] suggested that Ramadan intermittent fasting in obese men could decrease the level of IL‐6 and tumor necrosis factor‐alpha (TNF‐a), but not the CRP. The results are controversial. So far, few studies have been conducted to explore the changes and fluctuations of APRPs in fasting and nonfasting states. Hence, we speculated that the levels of APRPs could fluctuate in response to dietary intake and be associated with the duration of fasting.

In this study, we utilized a standardized experimental model (OGTT) to observe the changes in APRPs during fasting and at different time points after a standardized diet. All subjects fasted for 12 h after dinner from the previous day. Blood samples were collected the next day during fasting (8:00 am, T0), 30 min (8:30 am, T1), 60 min (9:00 am, T2), 120 min (10:00 am, T3), and 180 min (11:00 am, T4) after 75 g glucose intake. All blood samples were tested for APRPs, including CRP, IL‐6, procalcitonin (PCT), transferrin (TRF), prealbumin (PA), and ceruloplasmin (CER). By comparing with the levels in the fasting state, the changes of APRPs at different time points were explored. In addition, percentage bias was calculated and Bland–Altman analysis was performed to further assess clinical variability for APRPs.

## 2 Materials and Methods

### 2.1 Subjects

Subjects were enrolled to participate in the experiment with strict criteria. The inclusion criteria were established as follows: (1) live with a regular diet, in line with the local life habits; (2) no smoking history; (3) no drugs or alcohol were used for 2 weeks prior to the experiment; (4) no kidney, liver, or cardiovascular disease; (5) no physical impairment, mobility impairment, and no mental illnesses; (6) no blood‐related diseases, immune disease, or tumors. Ultimately, 50 subjects were enrolled, comprising 25 males and 25 females. Among them, 23 subjects were in the younger group (≤ 50 years) and 27 were in the older group (> 50 years). This study was approved by the Ethics Committee of the First Affiliated Hospital of Nanjing Medical University (2023‐SR‐374).

### 2.2 Study Design

In our analysis, 75 g of glucose was treated as the standardized diet according to the requirement of the clinical OGTT test. All subjects were required to fast after 8 pm the day before the experiment and fast for 12 h until 8 a.m. the next day. 3 mL of elbow venous blood was drawn from each subject and centrifuged for 5 min at a speed of 3000 rpm to isolate serum for detection. In total, 250 blood samples were obtained. During the experiment, all subjects fully complied with the requirements of the OGTT experiment: (1) fasting for 12 h after dinner, no tea or caffeinated beverages, and no strenuous exercise the day before the OGTT test; (2) normal physical activity, normal diet; (3) drugs such as thiazide diuretics, beta‐receptor antagonists, and glucocorticoids were avoided for three consecutive days before the test; (4) during the examination, subjects needed to be fasting, water prohibition, no smoking, sitting quietly, avoiding excessive activities; (5) 75 g of anhydrous glucose was dissolved in 250–300 mL of water and consumed in 5 min for each subject.

### 2.3 Laboratory Parameter Analysis

A total of six APRPs were analyzed in this study: CRP, PCT, IL‐6, TRF, PA, and CER. Six APRPs were measured for each subject at five time points. The Roche Cobas 602 ECL analyzer (Switzerland) was utilized to detect the level of PCT and IL‐6. The Beckman Coulter AU5800 automatic biochemical analyzer (American) was used to detect the level of CER. The Beckman Coulter IMMAGE 800 Specific Protein analyzer (American) was used to detect the level of CRP, TRF, and PA. All tests were performed under qualified quality control conditions.

### 2.4 Bland–Altman Analysis

Bland–Altman analysis is a widely used analytical tool in medical and scientific research, providing an intuitive visualization of bias and measurement error. It enables the assessment of agreement between two measurements by plotting the differences in their results, thereby identifying potential discrepancies and deviations [13, 14, 15]. In our study, Bland–Altman plots were utilized to effectively and visually demonstrate the bias in APRPs level fluctuations following dietary intervention. The percentage bias was analyzed by using the formula: (C_2_‐C_1_)/C_1_ × 100%. C_1_ represents the fasting concentration of the APRPs, and C_2_ represents the concentration of the APRPs at different times (30, 60, 120, and 180 min).

### 2.5 In Vitro Glucose Interference Assay

In this study, since significant fluctuations in serum glucose concentrations were observed following glucose intake, the glucose interference experiment was subsequently designed and conducted to investigate whether the changes affected the results. Briefly, we additionally selected blood samples with different concentrations of these APRPs during the OGTT. The fetal bovine serum (FBS) and glucose powder were utilized to prepare a 30 mmol/L high glucose solution. Then, the serum samples were diluted 1:1 with the high glucose solution (Solution 1) or diluted directly 1:1 with FBS (Solution 2). In the following analysis, the difference between the APRPs in Solution 1 and Solution 2 was compared. The bias between the two was calculated using the following formula: (C_Solution1_−C_Solution2_)/C_Solution2_ × 100%. C_Solution1_ and C_Solution2_ represent the concentration of the APRPs in solution 1 and solution 2, respectively. In addition, we also explored the effects of different concentrations of glucose solutions (50, 25, 12.5, 6.25 mmol/L) on the levels of APRP assays in serum samples. All tests were repeated three times.

In addition, to further investigate the potential associations between APRPs and blood glucose‐related factors, we collected clinical data from a cohort of 1000 patients examined in hospitals between January 1, 2020, and January 1, 2023. Spearman's rank correlation analysis was performed to elucidate the relationships among fasting glucose levels, insulin levels, C‐peptide levels, and the six APRPs.

### 2.6 Statistical Analysis

R 4.4.2 and GraphPad Prism 10.3.1 software were utilized for statistical analysis. The normally distributed data were expressed as mean ± standard deviation (M ± SD) and the one‐way analysis of variance was used to compare the statistical significance. Otherwise, data were expressed as median (quartile): M (P25, P75) and the Kruskal–Wallis one‐way analysis of variance was used to compare the statistical significance. Percentage bias (%) was calculated, and the Bland–Altman plots were plotted to show the difference of APRPs at different time points compared to the fasting one. Spearman analysis was used to analyze the correlation between serum glucose, insulin, and C‐peptide levels and six APRPs. *p* < 0.05 was considered statistically significant.

In addition, according to the total allowable error (TEa) of the Chinese external quality assessment (EQA) standard, the clinical maximum acceptable bias for each test was determined in this study. The details were as follows: 8.33% for CRP, IL‐6, PCT, PA, and TRF; 10% for CER. Additionally, current month laboratory quality control variable coefficients (CVs) were used to assess laboratory variability. The CVs were as follows: 7.39% (Level 1) and 7.71% (Level 2) for CRP; 4.6% and 3.15% for IL‐6; 4.55% and 4.21% for PCT; 7.26% and 7.3% for PA; 7.83% and 7.72% for TRF; 3.33% and 5.26% for CER.

## 3 Results

### 3.1 Clinical Characteristics of the Subjects

**FIGURE 1:**
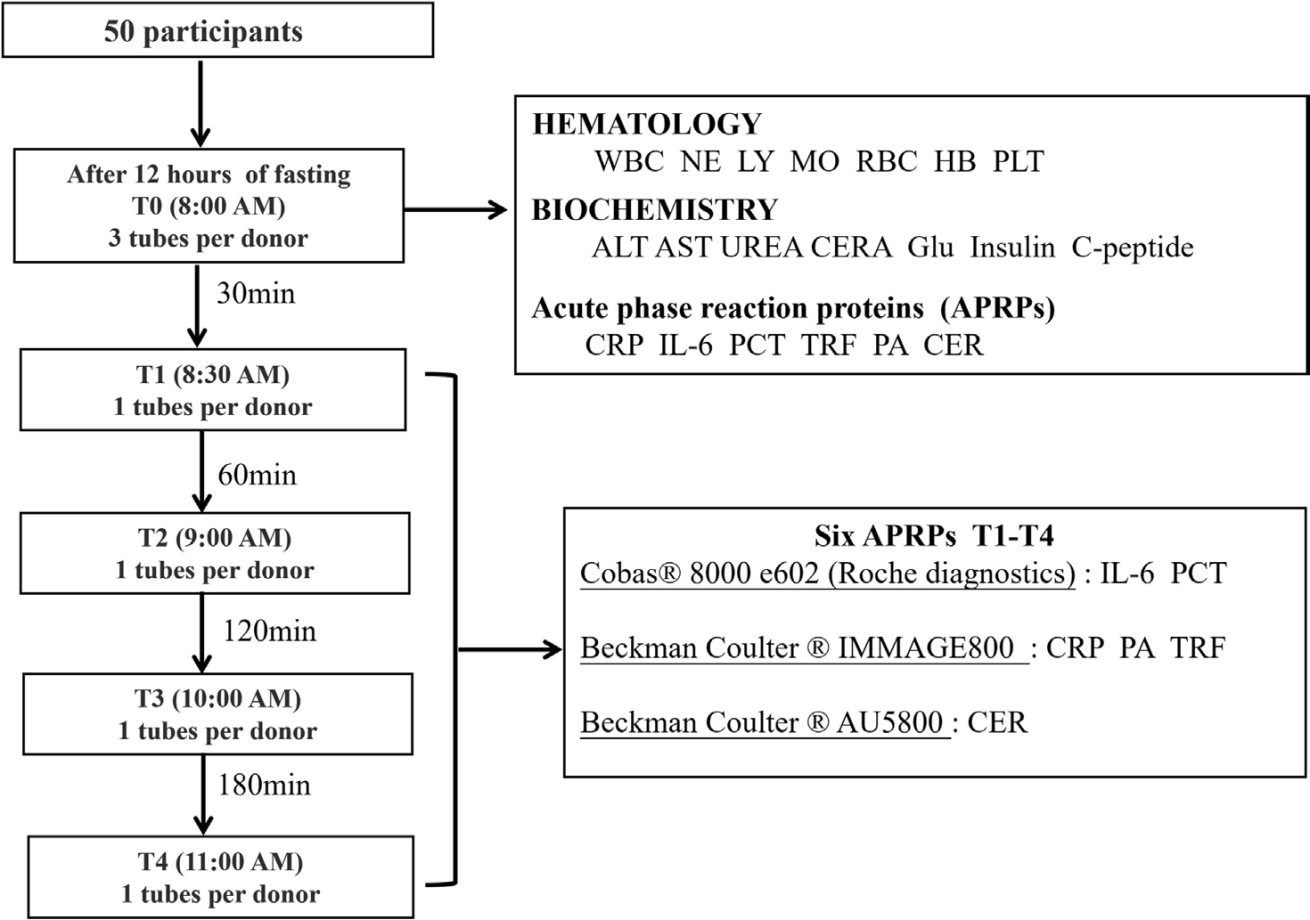
Basic flow chart of the experiment.

The basic flow of our experiment is illustrated in Figure 1, which provides a comprehensive overview of the study design and methodology. A total of 50 subjects were enrolled in our study, comprising 25 males and 25 females. Their age ranged from 23 to 77 years, with a median age of 53.5 years. Assessment of markers such as hematological parameters (leukocyte, erythrocyte, hemoglobin, and platelet), biochemical markers (ALT, AST, UREA, CERA, fasting blood‐glucose, insulin, and C‐peptide), and tumor‐related markers (AFP and CEA) revealed no abnormal signs among subjects involved in this study (Table 1).

**TABLE 1:** Clinical characteristics of the subjects in the study.

Characteristics	Laboratory results median (*Q*_1_, *Q*_3_)
Age(years)	53.50 (43.00, 61.75)
Fasting blood‐glucose (mmol/L)	6.04 (5.34, 7.89)
Fasting C‐peptide (mmol/L)	536.70 (360.72, 916.27)
Fasting insulin (mmol/L)	43.05 (19.95, 89.87)
AST (U/L)	21.60 (17.05, 30.00)
ALT (U/L)	19.45 (16.00, 41.75)
UREA (mmol/L)	5.29 (4.11, 6.49)
CREA (μmol/L)	58.05 (52.00, 69.95)
Leukocyte count (10^9/L)	6.19 (5.51, 7.27)
Neutrophil count (10^9/L)	3.79 (2.83, 4.72)
Lymphocyte count (10^9/L)	1.98 (1.48, 2.21)
Monocyte count (10^9/L)	0.40 (0.34, 0.51)
Erythrocyte count (10^12/L)	4.62 (4.23, 4.84)
Hemoglobin (g/L)	136.50 (128.00, 142.75)
Platelet (10^9/L)	224.50 (165.75, 283.75)
CEA (ng/mL)	2.42 (1.31, 3.40)
AFP (ng/mL)	2.49 (1.82, 3.21)

Abbreviations: AFP, alpha‐fetoprotein; ALT, alanine aminotransferase; AST, aspartate aminotransferase; CEA, carcinoembryonic antigen; CREA, creatinine; UREA: carbamide.

### 3.2 Changes in Serum APRPs in Fasting and After the Glucose Intake

**FIGURE 2:**
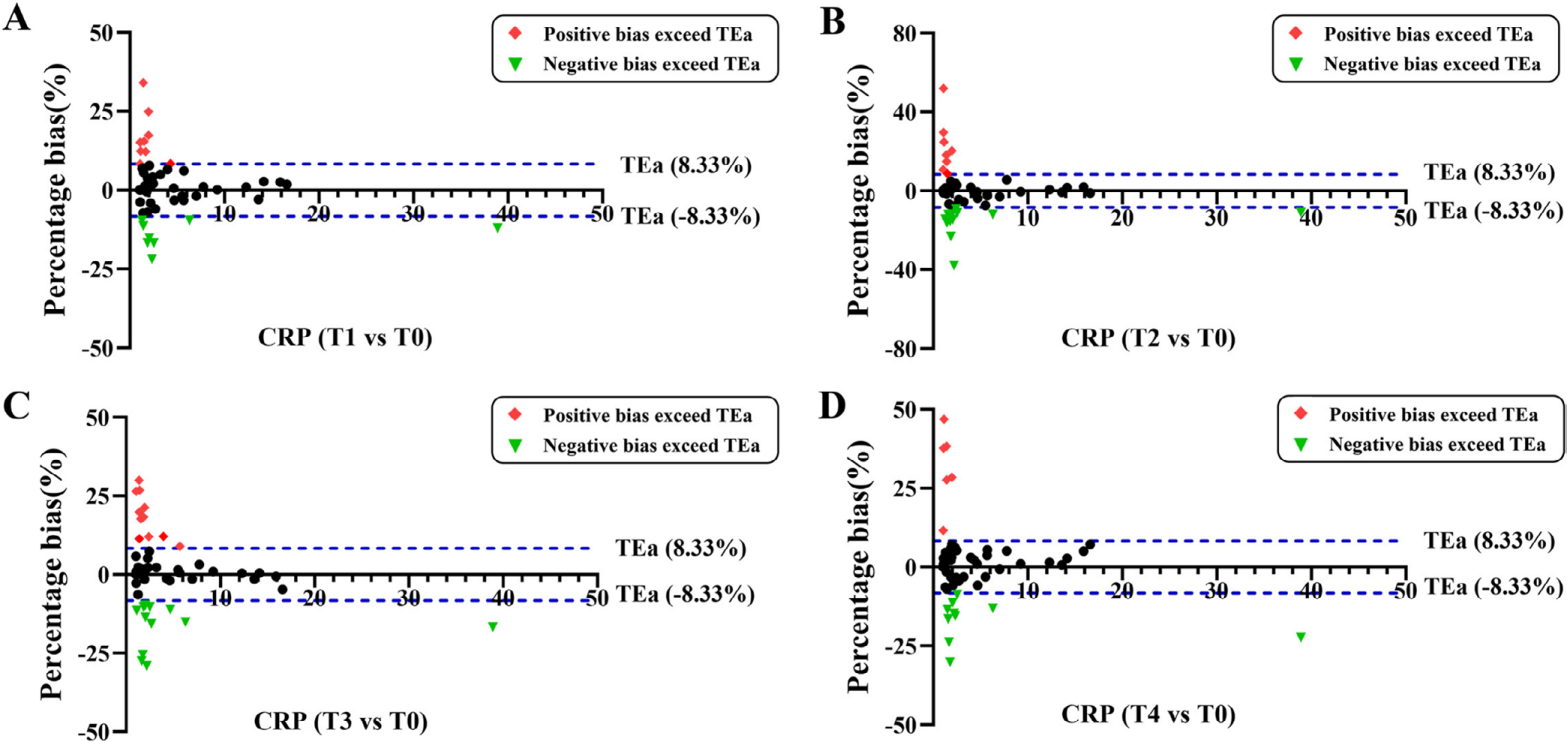
Clinical bias analysis for CRP compared with fasting in 50 subjects. The blue dashed lines are the total allowable error for medical laboratories. The green inverted triangle represents negative clinical bias beyond the TEa, and red rhombus dots represent positive clinical bias beyond 8.33%. Percentage bias was calculated in the 30, 60, 120, and 180 min compared to the fasting (A‐D).

Compared with the fasting state, the clinical changes and fluctuations of APRPs at different times were observed. For CRP, there were 17 (34%) of 50 subjects whose bias exceeded the clinical maximum acceptable range at the time T1 compared to T0. Of these, nine subjects exhibited positive bias changes and eight subjects showed negative bias (Figure 2A). At time T2, a total of 21 (42%) subjects demonstrated clinically unacceptable bias, with 8 showing positive bias and 13 exhibiting negative bias (Figure 2B). In addition, at time T3 and T4, 23 (46%) and 16 (32%) subjects, respectively, had the change in clinical bias of more than TEa of Chinese EQA standard (Figure 2C,D).

**FIGURE 3:**
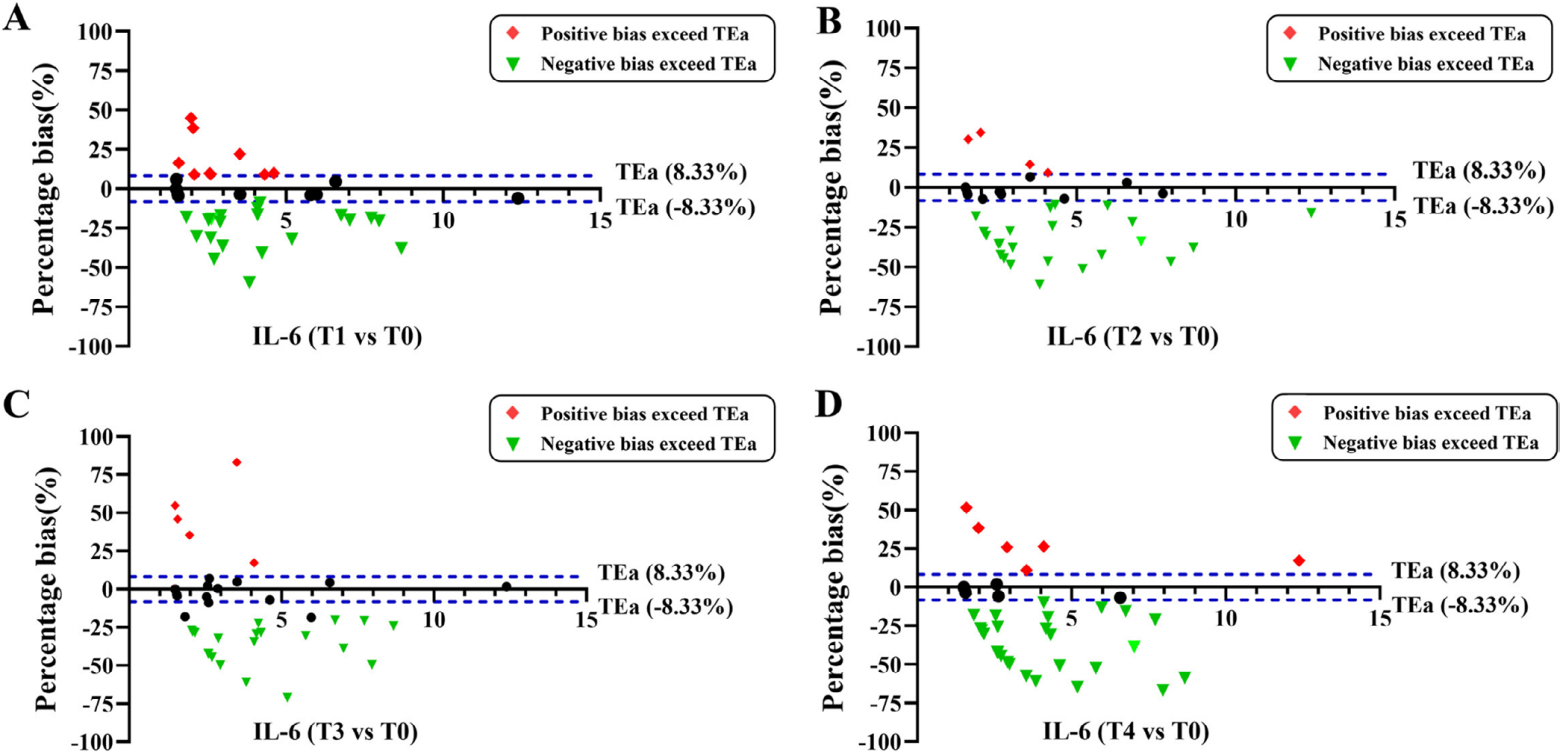
Clinical bias analysis for IL‐6 compared with fasting in 50 subjects. The blue dashed lines indicate the maximum acceptable bias thresholds for medical laboratory measurements. The green inverted triangle denotes instances of negative clinical bias exceeding the clinically acceptable range, while the red rhombus dots represent positive clinical bias surpassing the 8.33% threshold. Percentage bias was calculated at time intervals (30, 60, 120, and 180 min) relative to fasting baseline measurements, as illustrated in panels A through D.

As for IL‐6, comparable findings were observed (Figure 3). In comparison to the fasting state, the clinical difference beyond the maximum acceptable range was found in 30 (60%) subjects at T1 time. Specifically, positive differences were noted in 10 individuals, while negative differences occurred in 20 individuals (Figure 3A). In addition, 27 (54%), 28 (56%), and 32 (64%) subjects displayed clinical differences greater than TEa at time T2 (Figure 3B), T3 (Figure 3C), and T4 (Figure 3D), respectively, showing the unacceptable bias.

Regarding the PCT, there were 14 (28%), 12 (24%), 14 (28%), and 13 (26%) subjects who had clinical bias exceeding the maximum permissible range at the T1–T4 time points (Supporting Information Figures S1 and 4). As for TRF, 18 (36%), 10 (20%), 13 (26%), and 19 (38%) exceeded the clinical maximum acceptable bias at T1–T4 time (Figures S2 and 4). As for PA, at times T1, T2, T3, and T4, there were 12 (24%), 11 (22%), 13 (26%), and 17 (34%) subjects, respectively, who overstepped clinical permissible maximum bias (Figures S3 and 4). Regarding CER, 15 (30%), 14 (28%), 15 (30%), and 16 (32%) exceeded clinical acceptable maximum deviations during T1–T4 (Figures S4 and 4).

**FIGURE 4:**
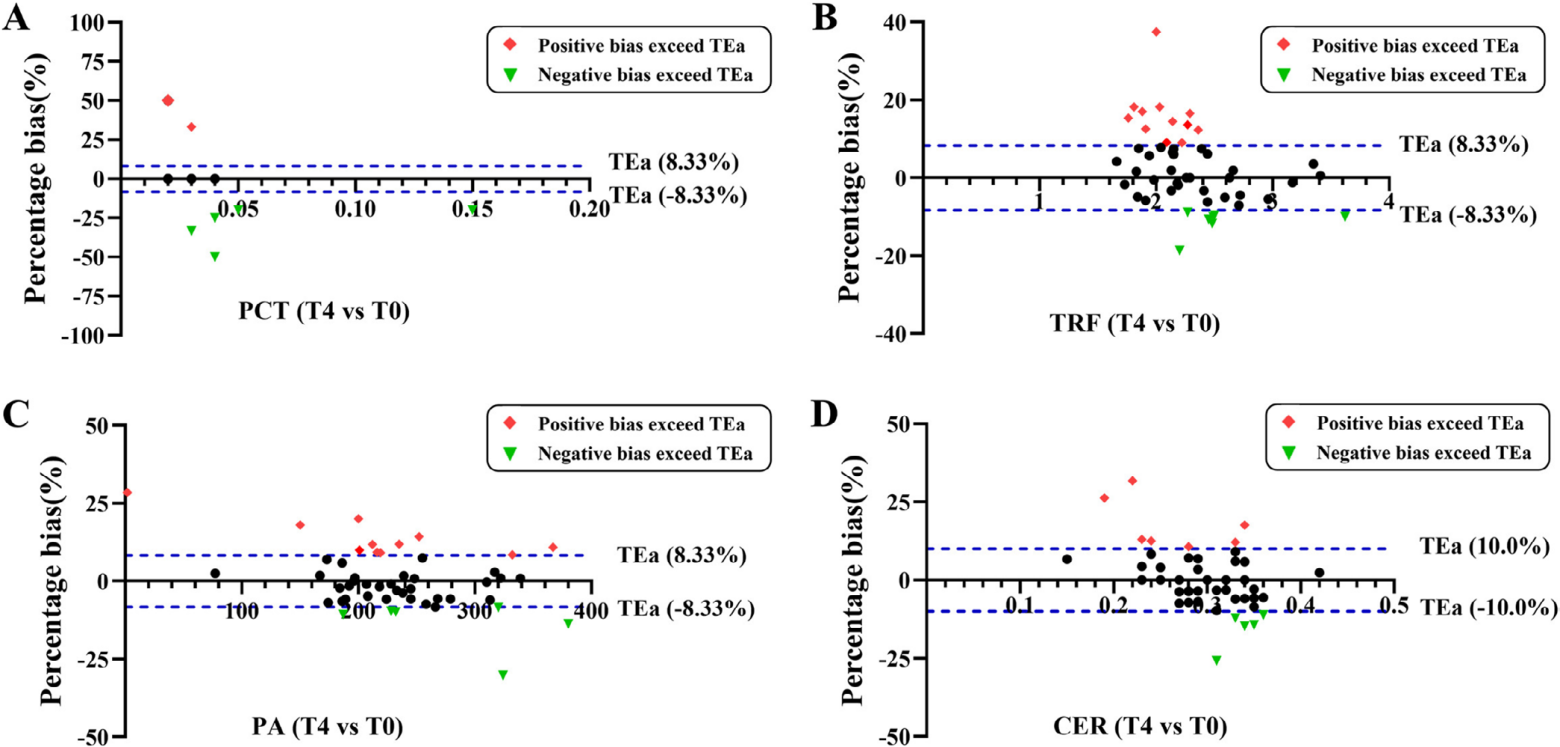
Bias analysis for PCT, TRF, PA, and CER at time T4 compared with fasting in 50 subjects. The blue dashed lines demarcate the limits of permissible bias for clinical laboratory measurements. Data points marked in green inverted triangles indicate negative clinical bias values that exceed the predefined acceptable range, whereas red rhombus data points correspond to positive clinical bias values greater than 8.33%. The percentage bias was evaluated at T4 (180 min) relative to fasting baseline values, with the corresponding results presented in panels A (PCT), B (TRF), C (PA), and D (CER).

### 3.3 Subgroup Analysis for Age and Gender

To investigate whether the variations in APRPs were influenced by factors such as age and gender, subgroup analysis was conducted. Among the 50 subjects, 22 were aged 50 years or younger, while 28 were older than 50 years. For CRP in the younger group, the number of subjects whose changes at T1, T2, T3, and T4 exceeded the clinically permissible maximum error were 5 (9.09%), 7 (31.82%), 9 (40.91%), and 3 (13.64%), respectively (Table S1). In the older group, the corresponding numbers were 12 (42.86%), 14 (50%), 14 (50%), and 13 (46.43%) (Table S1). Chi‐square test results indicated no significant difference in the proportion of subjects exceeding TEa between the two groups (*p* = 0.593). Additionally, for IL‐6, PCT, TRF, PA, and CER, there were also no significant differences between age groups (all *p* values greater than 0.05, Table S1). These findings suggested that age was not the primary factor contributing to the observed bias.

In addition, of the 50 subjects, there were 25 men and 25 women. Chi‐square test results also showed that there was no significant difference between the two gender groups in the distribution of subjects over TEa from T1–T4 (*p* values were all greater than 0.05, Table S2).

### 3.4 Results of Glucose Interference Experiments

**FIGURE 5:**
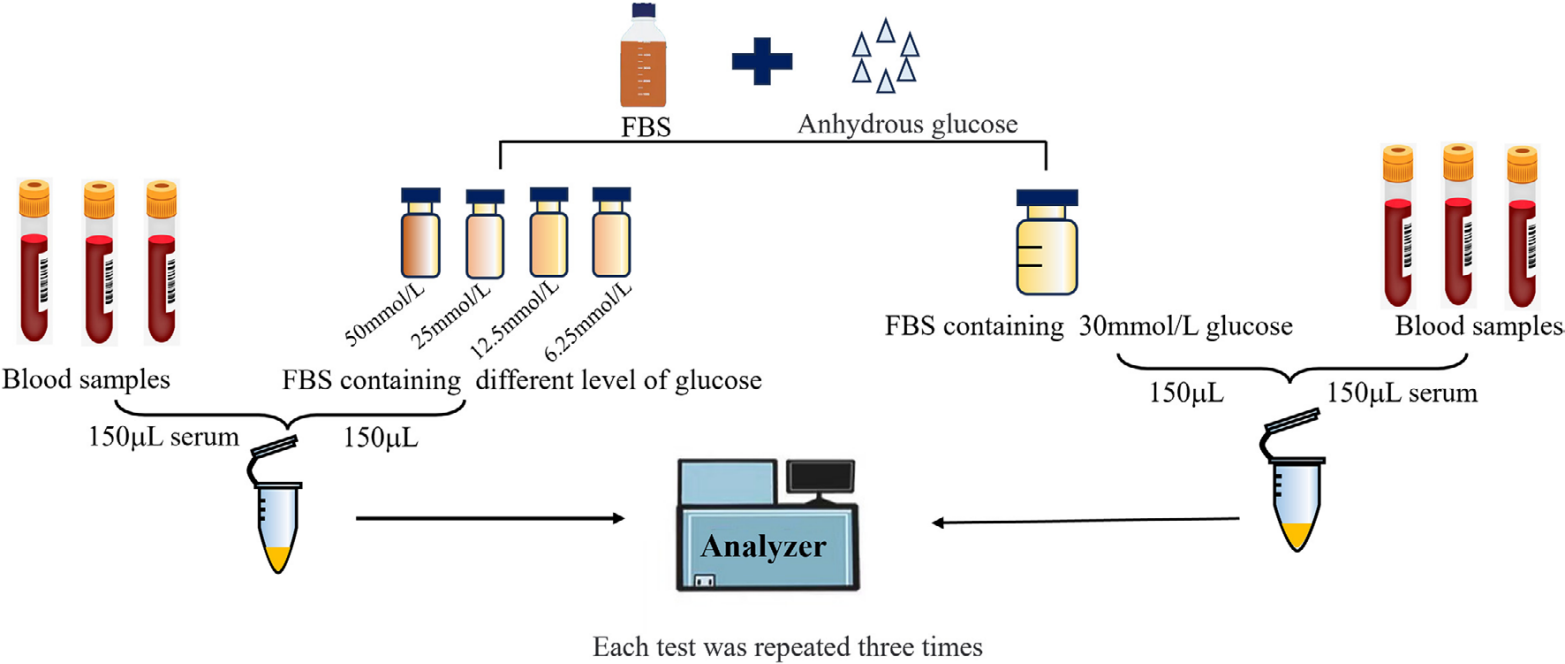
Basic flow chart of glucose interference experiments.

To comprehensively investigate whether fluctuations in serum glucose concentrations affect test results, the in vitro glucose interference study was conducted. The detailed experimental procedure is illustrated in Figure 5. Initially, serum samples with varying concentrations of APRPs were selected from specimens obtained during the experiment. Subsequently, we prepared an FBS solution and configured to contain a concentration of 30 mmol/L glucose. Meanwhile, the blood samples were also diluted 1:1 with the FBS solution. The results showed that the percentage bias did not exceed the maximum clinically permissible bias across all six PARPs (Table S3); this suggested that high levels of glucose did not cause the bias difference in APRPs levels.

**FIGURE 6:**
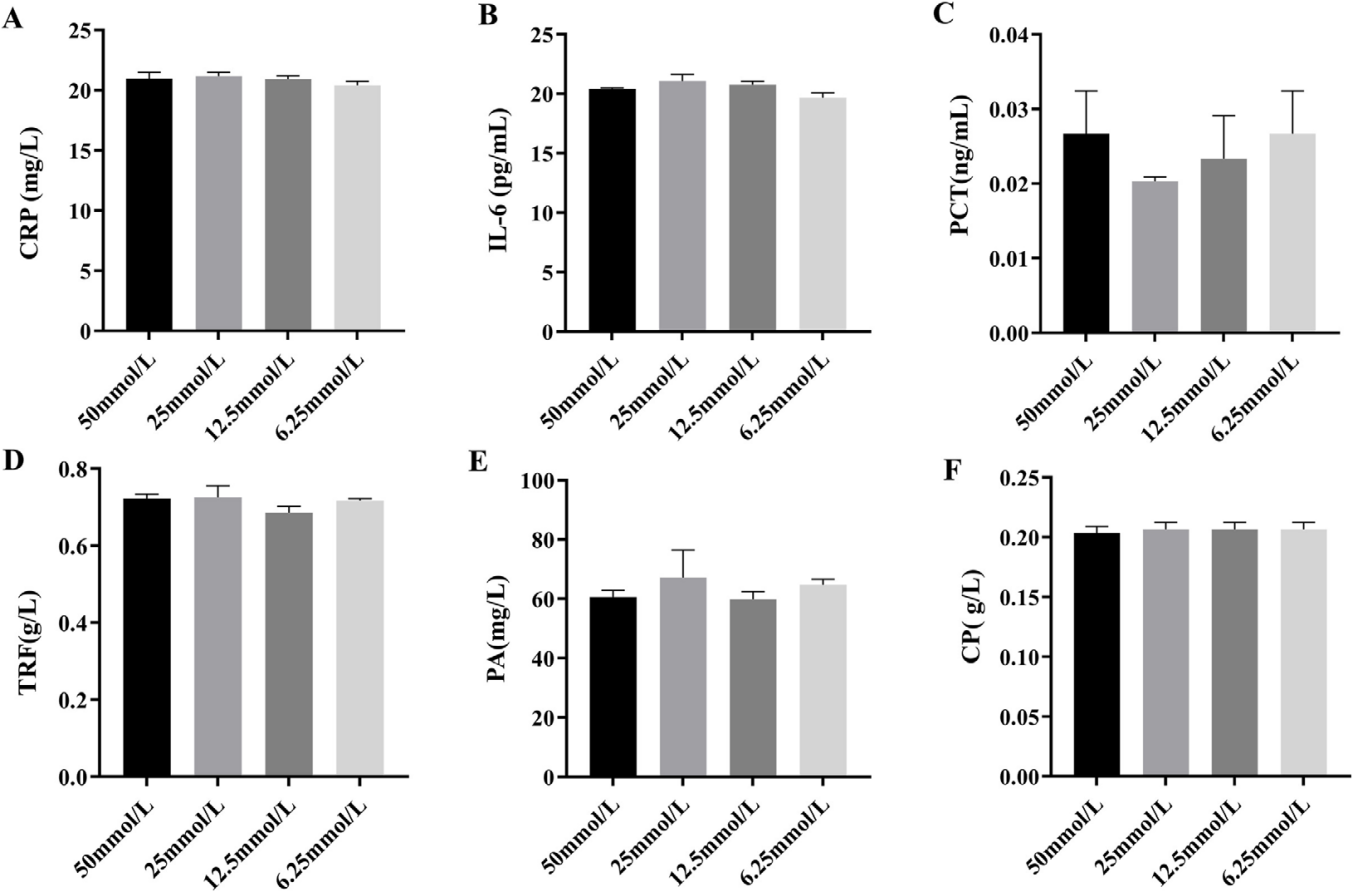
Results of glucose interference experiments. Different concentrations of glucose solutions were utilized to explore the effect of glucose changes on the results of acute‐phase reactive proteins. The results showed that different concentrations of glucose had no effect on CRP (A), IL‐6 (B), PCT (C), TRF (D), PA (E), and CP (F). *p* values greater than 0.05 for all groups (Kolmogorov–Smirnov).

In addition to examining the high glucose conditions, we expanded our investigation by assessing how various concentrations of glucose might influence six APRPs. The result is shown in Figure 6. For this part of the study, serum samples were mixed at an equal volume ratio (1:1) with solutions containing differing concentrations of glucose (50, 25, 12.5, and 6.25 mmol/L). Statistical analyses revealed that none of these different glucose concentrations had any significant effect on the results (all *p* > 0.05). These consistent findings indicated no interference from varying degrees of blood glucose levels when measuring clinical bias associated with APRPs.

### 3.5 Correlation Analysis

To fully explore whether there were significant associations between fasting glucose, insulin, and C‐peptide levels and the observed APRPs, we retrospectively collected data from a population of 1000 who underwent medical examinations at the health management center in our hospital. Spearman correlation analysis showed that there was no significant correlation between different levels of blood glucose and the APRPs in the population; the absolute value of correlation coefficients was all less than 0.1 (Figure S5). In addition, there were also no significant correlations between serum insulin, C‐peptide, and these APRPs in the observed population.

## 4 Discussion

There is currently no consensus regarding the necessity of fasting prior to the testing of APRPs. In clinical practice, APRPs are generally tested at random intervals. Furthermore, for the evaluation of disease progression, patients typically undergo multiple tests. Through the comparative analysis of alterations in APRPs, clinicians can make informed medical decisions. However, our study revealed discrepancies and fluctuations in APRPs levels between fasting and nonfasting states. This highlights the need to assess the reliability of random test results in reflecting the state of the patients. Plumelle et al. [16] provided standardized breakfast and lunch to 20 subjects to evaluate 77 laboratory indicators. The results revealed variations in 20 analytes, with fibrinogen, a type of APRPs, showing a significant change after lunch compared to the fasting state. In a study conducted by Lin et al. [17], the hs‐CRP levels changed significantly in coronary heart disease patients after a daily meal. The researchers believed that the nonfasting hs‐CRP level was better than the fasting hs‐CRP to evaluate the inflammatory status of patients. Therefore, in conjunction with our findings, clinicians should be aware of the differences caused by fasting and the time of sampling when performing fasting or nonfasting tests.

In this study, we utilized a standardized study model to analyze the APRPs changes in each subject. Blood samples were collected during fasting, 30, 60, 120, and 180 min after glucose intake. We found significant differences in APRPs test results at different times compared to fasting. In our analysis, we observed a diverse range of changes in these APRPs. Notably, for CRP and IL‐6, we identified clinically unacceptable bias in nearly half of the subjects compared to fasting levels. For CRP, 17, 21, 23, and 16 out of 50 subjects exceeded the maximum allowable error established by medical laboratories after 30‐, 60‐, 120‐, and 180‐min glucose intake. A previous report had suggested clinically significant variations in CRP levels 4 h after dietary intake; although the increase was not statistically significant by Mann–Whitney test, the researchers have suggested that the laboratory management should standardize the fasting time for all laboratory tests [5]. Moreover, Wirth et al. [6] indicated that each additional hour of overnight fasting significantly correlated with fluctuating CRP levels. Notably, a study by Lin et al. [17] reported that CRP levels were significantly elevated at 2 h and 4 h after a normal breakfast compared to fasting levels, with statistically significant differences. In contrast, our study showed inconsistent fluctuations (positive and negative bias) in CRP after standardized glucose intake. Nevertheless, these findings, which supported that CRP concentrations were influenced by fasting and fasting time, still hold. As for IL‐6, there were 27, 28, and 32 subjects with bias beyond the maximum clinically acceptable range after 60, 120, and 180 min, respectively. Previous studies have shown that Ramadan intermittent fasting over a period of 30 days could reduce IL‐6 levels [12]. According to Moro et al. [18], a 4‐week time‐restricted feeding (TRE, a type of IF) could also reduce IL‐6 levels in athletes. Research on animal models indicated that IF could suppress the nuclear factor‐κB (NF‐κB) signaling cascade [19]. As a crucial transcriptional regulator of inflammation, NF‐κB played a pivotal role in controlling the production of various inflammatory mediators, including IL‐1β, IL‐6, and TNF‐α. These published studies pointed out that IL‐6 levels were reduced during prolonged fasting or IF. In our investigation, the subjects underwent only 12 h of overnight fasting, which was more representative of typical clinical scenarios involving patients. Similar to CRP, our results showed obvious fluctuations in the levels of IL‐6 after diet, nearly half of the subjects exceeding the clinically maximum acceptable bias.

As for PCT, which served as a biomarker for infection and was utilized across diverse clinical settings, including primary care, emergency departments, and intensive care units [20]. In our study, all 50 subjects exhibited low PCT levels, ranging from 0.02 to 0.04 ng/mL, which suggested the absence of infection and inflammation. So far, limited research has been conducted on the relationship between dietary factors and PCT levels. According to Schaible [21], the interaction between the state of the body's nutrition and the immune system could contribute to the risk of infection and indirectly affect the levels of inflammatory markers. Regarding TRF, it is a glycoprotein synthesized by the liver and is primarily responsible for iron transport and metabolism. Studies have shown that TRF is an important nutrition‐related biomarker commonly used to assess nutritional status in patients with protein–energy malnutrition (PEM) and chronic diseases [22, 23]. Previous studies have reported that changes in TRF was associated with changes in insulin resistance [24]. A randomized controlled trial experiment showed variational TRF levels after fasting administration [18]. These studies seemed to show that TRF levels were influenced by nutritional and fasting status. In our study, we observed significant fluctuations in TRF levels in 19 subjects. These findings suggested that TRF levels were influenced by both nutritional and fasting states. As for PA, a negative acute‐phase reactive protein, exhibits decreased serum levels during inflammatory states and diseases. With its rapid metabolism and high sensitivity to changes in nutritional status, PA serves as an effective biomarker for assessing early alterations in a patient's nutritional condition [25, 26]. Evidence has shown that PA levels were significantly affected by dietary intake [27]. Changes in PA levels may need to be evaluated in combination with inflammatory markers and nutrient intake. The results of our data analysis showed that 12,11,13, and 17 subjects, respectively, had changes in clinical bias at T1–T4 that exceeded the limits of TEa compared to T0. About serum CER, a copper‐containing glycoprotein synthesized primarily by the liver, its main functions include the transport of copper and the regulation of iron metabolism. According to Schulpis et al. [28], analysis of data after 60 days of dietary control found that serum CER levels were significantly associated with dietary factors. In our analysis, we observed fluctuations in the CRE levels at different times after a standardized dietary.

In our analysis, to further explore whether there were correlations between changes and age or gender, subgroup analyses were performed. The results of the chi‐square test showed that there was no significant association. In the physiological state, glucose is the main energy source for most tissues. After a meal, glucose is generated and converted into energy. During prolonged fasting, the glucose concentration in the blood will decrease, and glycolysis will be inhibited. Glycogen reserves in the liver are depleted, and gluconeogenesis is initiated [29]. In this study, due to the glucose intake, there were significant differences in blood glucose levels among the individuals. We initially hypothesized that these changes might be attributed to variations in blood glucose levels. Consequently, we conducted the experiment to investigate the effects of glucose interference. On one side, the alterations in APRPs across various blood samples were explored by introducing a 30 mmol/L glucose solution to the serum; however, no significant changes in APRP levels were detected at high glucose concentrations, indicating that elevated glucose does not influence APRP levels. In addition, the effects of glucose solutions with varying concentration gradients were also examined. The results indicated no substantial changes following different degrees of glucose interference. Thus, we concluded that these variations in APRPs were independent of blood glucose fluctuations. Subsequently, we further analyzed the correlation between fasting blood glucose, insulin, and C‐peptide levels and APRPs by analyzing 1000 individuals who were examined in hospitals. Our analysis revealed no significant correlations between APRP levels and those of glucose, insulin, or C‐peptide. This further supported the notion that alterations in APRPs were not attributable to glucose‐related factors.

Researches have shown that the response of healthy cells to fasting is evolutionarily conserved, which plays a role in protecting cells and prolonging life span [30, 31, 32]. A recent review indicates that fasting exerts its beneficial effects on health through multiple mechanisms. It activates the AMP‐activated protein kinase (AMPK) pathway, which enhances autophagy and mitigates inflammation by inhibiting the mammalian target of rapamycin (mTOR) pathway [33]. Besides, the insulin‐like growth factor 1 (IGF1) signaling cascade is a key signaling pathway that mediates the effects of fasting at the cellular level. During fasting, the IGF1 levels and downstream signaling are reduced; the level of AKT‐mediated repression of mammalian FOXO transcription factors was down regulated, leading to the activation of enzymes such as heme oxygenase 1 (HO1), superoxide dismutase (SOD), and catalase, which exert antioxidant activity and protection [34, 35]. These alterations are accompanied by cellular and molecular adaptations of neural networks in the brain, which may improve neural network functionality and make resistance to stress, injury, and disease [36]. Additionally, fasting can modify the gut microbiota composition, promoting beneficial bacterial populations that contribute to systemic anti‐inflammatory responses, ultimately aiding in the prevention and management of diseases such as metabolic disorders, neurodegenerative conditions, and autoimmune diseases [33, 37, 38]. Particularly in cancer patients, fasting was proved to force healthy cells into a slow division and highly protective mode, protecting them from toxic damage from anticancer drugs [35, 39, 40]. In our analysis, although the molecular mechanisms underlying these biased fluctuations remain unexplored, our research proposed preliminary evidence pointing toward dietary influences as potential factors. Our analysis suggested that age, gender, and blood glucose levels were not responsible for the changes in APRPs. In subsequent research, we aim to delve into specific metabolic pathways mentioned above, such as the NF‐κB, AMPK, IGF1 signaling cascade, and nutritional alterations to elucidate the precise molecular mechanisms governing APRPs fluctuations postdietary intervention.

Although our analysis revealed the fluctuation and changes of APRPs in fasting and nonfasting states, several limitations were worth considering. Firstly, we used a standardized OGTT experimental model, which was conducive to the standardization of the experiment but does not necessarily fully reflect the results of normal dietary patterns. Especially for emergency and inpatient patients, there is great heterogeneity in daily diet. Nevertheless, clinicians need to pay attention to the changes and fluctuations of APRPs caused by dietary factors. Secondly, in this study, we only observed APRPs changes after 180 min of diet. Future studies should consider extending the observation time to evaluate these changes more fully. Thirdly, although our analysis excluded age, gender, and blood glucose concentration as contributing factors to the variations, further research is essential to elucidate the underlying mechanisms driving these discrepancies. It is of great significance to clarify whether these variations owe to the metabolic pathways, hormonal regulation, and inflammatory processes, and this understanding will deepen our knowledge of APRPs dynamics in clinical contexts.

In conclusion, our study provided initial insights into differences observed between these APRPs under fasting and nonfasting conditions: the fluctuations in APRPs among subjects following dietary intake were not consistent directional changes. When exploring potential confounding factors, we found that age, gender, and variations in blood glucose concentration were not significant contributors to the observed fluctuations in APRPs. The specific molecular mechanisms underlying these observations remain to be further elucidated through future research. Based on these findings, we strongly recommend that clinical assessments should be conducted under consistent conditions, either in the fasting state or at a consistent time interval after meals, to minimize variability induced by dietary factors.

## Ethics Statement

The human ethics involved in this study were approved by the Ethics Committee of the First Affiliated Hospital of Nanjing Medical University (2023‐SR‐374). Written informed consent for participation was not required for this study in accordance with the national legislation and the institutional requirements.

## Conflicts of Interest

The authors declare no conflicts of interest.

## Supporting information


**FIGURE S1.** Bias analysis for PCT compared with fasting in 50 subjects.
The maximum allowable bias thresholds are represented by blue dashed lines. Clinical deviations are indicated by colored data points, with green markers designating negative bias values outside the acceptable clinical range and red markers identifying positive bias exceeding the 8.33% threshold. Bias analysis was performed at standardized time intervals (30, 60, and 120 min) postbaseline.

**FIGURE S2.** Bias analysis for TRF compared with fasting in 50 subjects from T1–T3.
The blue dashed lines indicate the total allowable error (TEa). Negative clinical bias exceeding the TEa is represented by green inverted triangles, while red rhombus dots denote positive clinical bias surpassing 8.33%. Percentage bias was calculated at 30, 60, and 120 min and compared to fasting values (A–C).

**FIGURE S3.** Bias analysis for PA compared with fasting in 50 subjects from T1–T3.
The blue dashed lines depict the total allowable error (TEa). Green inverted triangles signify negative clinical bias exceeding the TEa, whereas red rhombus markers indicate positive clinical bias beyond TEa. The percentage bias was computed at intervals of 30, 60, and 120 min and compared to fasting measurements (A–C).

**FIGURE S4.** Bias analysis for CER compared with fasting in 50 subjects from T1–T3.
The blue dashed lines represent the total allowable error (TEa). Negative clinical bias exceeding the TEa is marked by green inverted triangles, while red rhombus symbols highlight positive clinical bias beyond the TEa. Percentage bias was calculated at 30‐, 60‐, and 120‐min intervals and compared to fasting measurements (A–C).

**FIGURE S5.** Correlation analysis.
Spearman correlation analysis was performed to analyze the relationship between blood glucose, insulin, C‐peptide, and the acute‐phase reactive proteins. The numbers in the figure represent the values of the correlation coefficients by spearman analysis.

**TABLE S1.** Subgroup analyses of age in 50 subjects.

**TABLE S2.** Subgroup analyses for gender.

**TABLE S3.** Results of glucose interference experiments.

## Data Availability

The data that support the findings of this study are available from the corresponding author upon reasonable request.
