# Supplementary figures and images for: Fluctuations and Changes in Acute Phase Reactive Proteins in Fasting and Nonfasting States
Source: J Clin Lab Anal. 2025 May 10;39(12):e70052. doi: 10.1002/jcla.70052 (PMC12179803; doi:10.1002/jcla.70052)

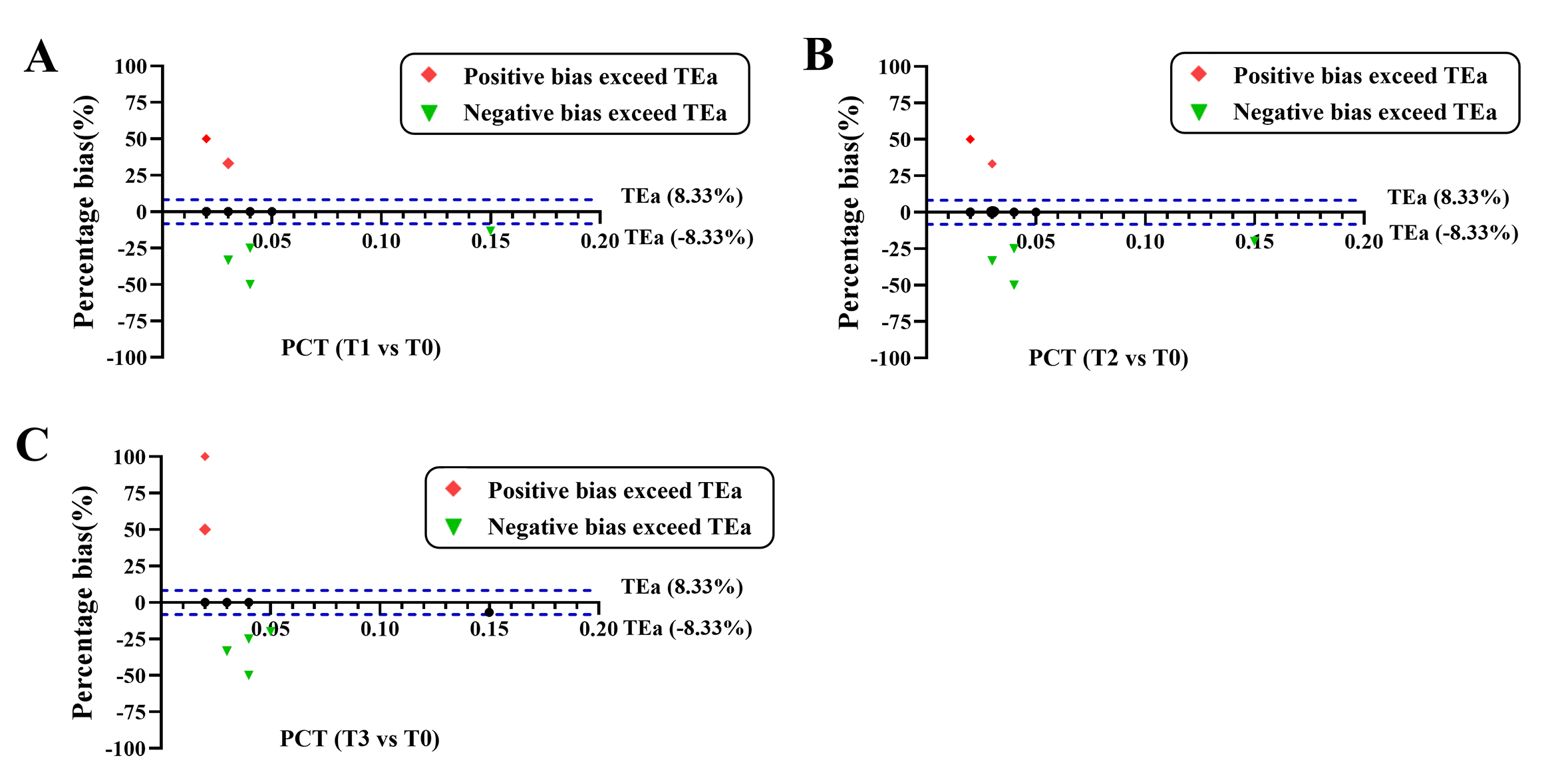

Supplement: Supplementary file 1 — FIGURE S1. Bias analysis for PCT compared with fasting in 50 subjects. The maximum allowable bias thresholds are represented by blue dashed lines. Clinical deviations are indicated by colored data points, with green markers designating negative bias values outside the acceptable clinical range and red markers identifying positive bias exceeding the 8.33% threshold. Bias analysis was performed at standardized time intervals (30, 60, and 120 min) postbaseline. [file JCLA-39-e70052-s005.tif]

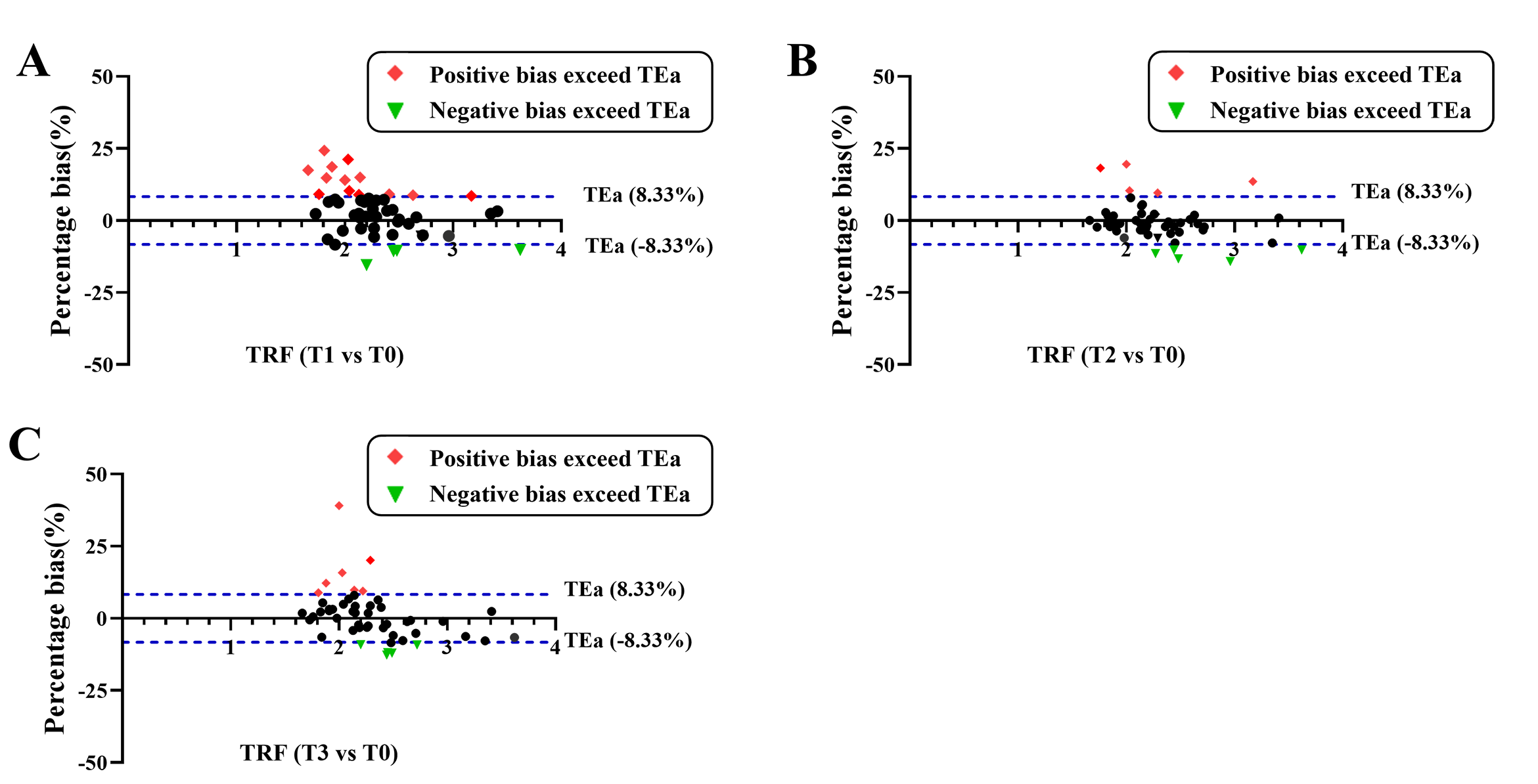

Supplement: Supplementary file 2 — FIGURE S2. Bias analysis for TRF compared with fasting in 50 subjects from T1–T3. The blue dashed lines indicate the total allowable error (TEa). Negative clinical bias exceeding the TEa is represented by green inverted triangles, while red rhombus dots denote positive clinical bias surpassing 8.33%. Percentage bias was calculated at 30, 60, and 120 min and compared to fasting values (A–C). [file JCLA-39-e70052-s002.tif]

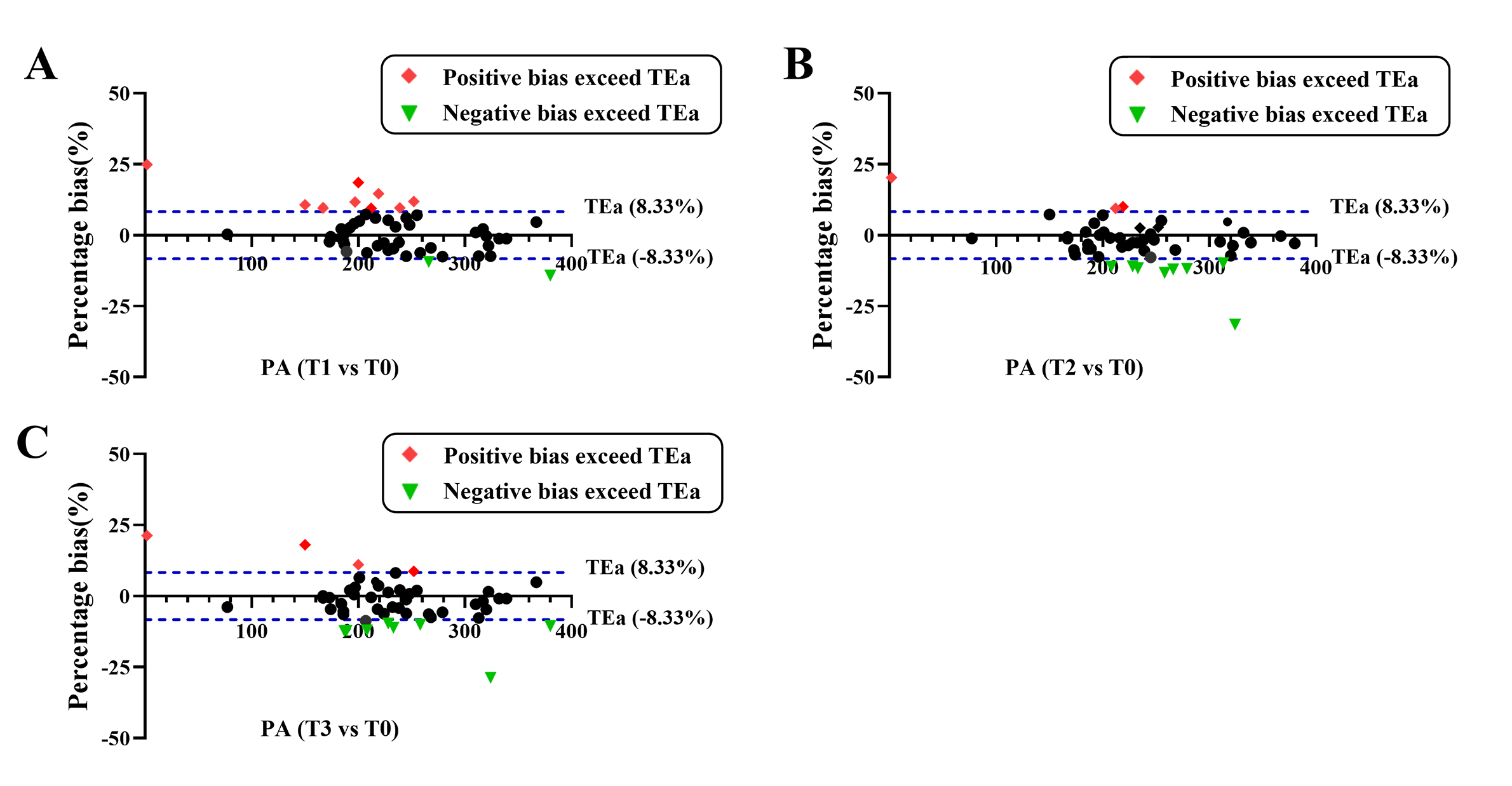

Supplement: Supplementary file 3 — FIGURE S3. Bias analysis for PA compared with fasting in 50 subjects from T1–T3. The blue dashed lines depict the total allowable error (TEa). Green inverted triangles signify negative clinical bias exceeding the TEa, whereas red rhombus markers indicate positive clinical bias beyond TEa. The percentage bias was computed at intervals of 30, 60, and 120 min and compared to fasting measurements (A–C). [file JCLA-39-e70052-s007.tif]

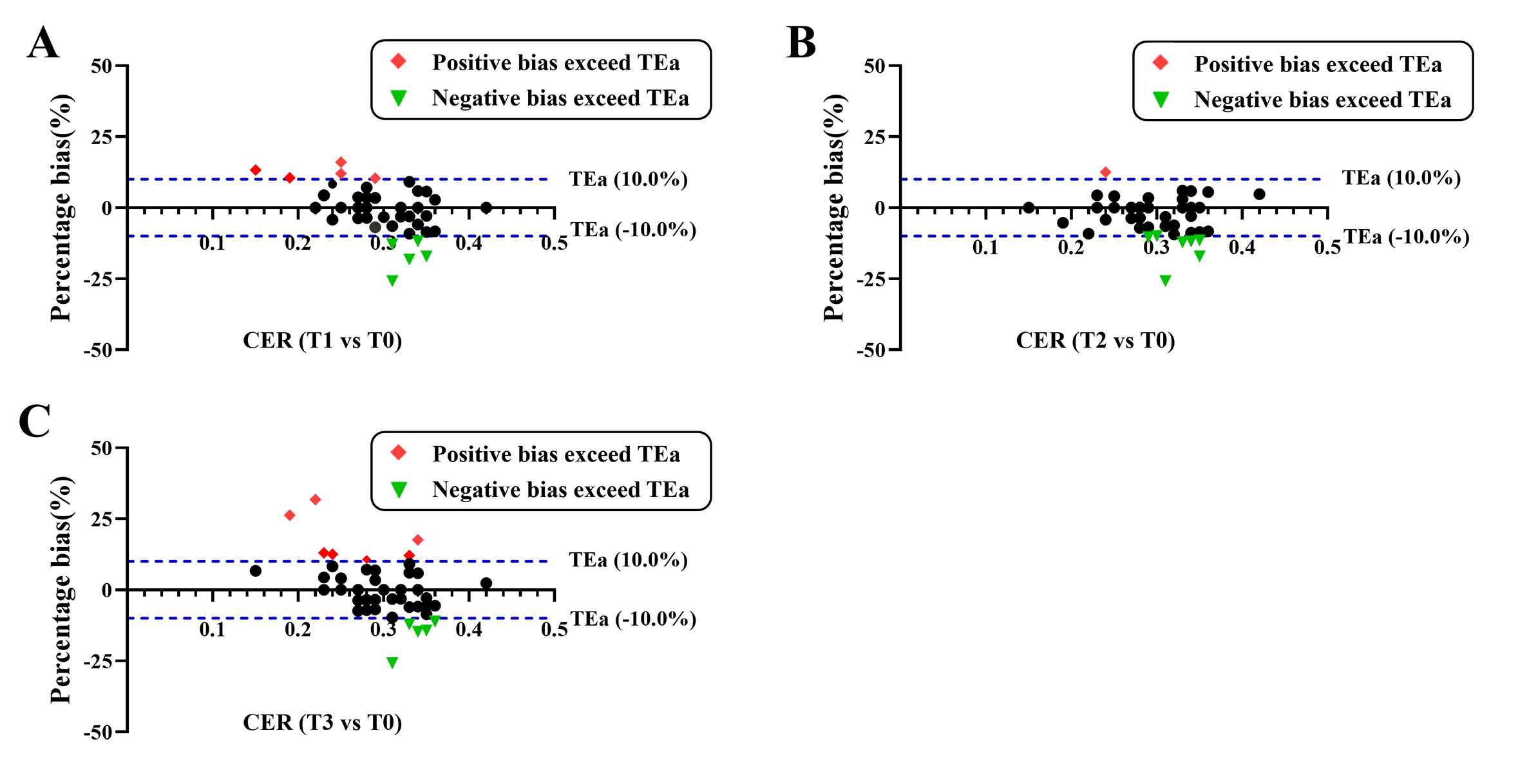

Supplement: Supplementary file 4 — FIGURE S4. Bias analysis for CER compared with fasting in 50 subjects from T1–T3. The blue dashed lines represent the total allowable error (TEa). Negative clinical bias exceeding the TEa is marked by green inverted triangles, while red rhombus symbols highlight positive clinical bias beyond the TEa. Percentage bias was calculated at 30‐, 60‐, and 120‐min intervals and compared to fasting measurements (A–C). [file JCLA-39-e70052-s004.tif]

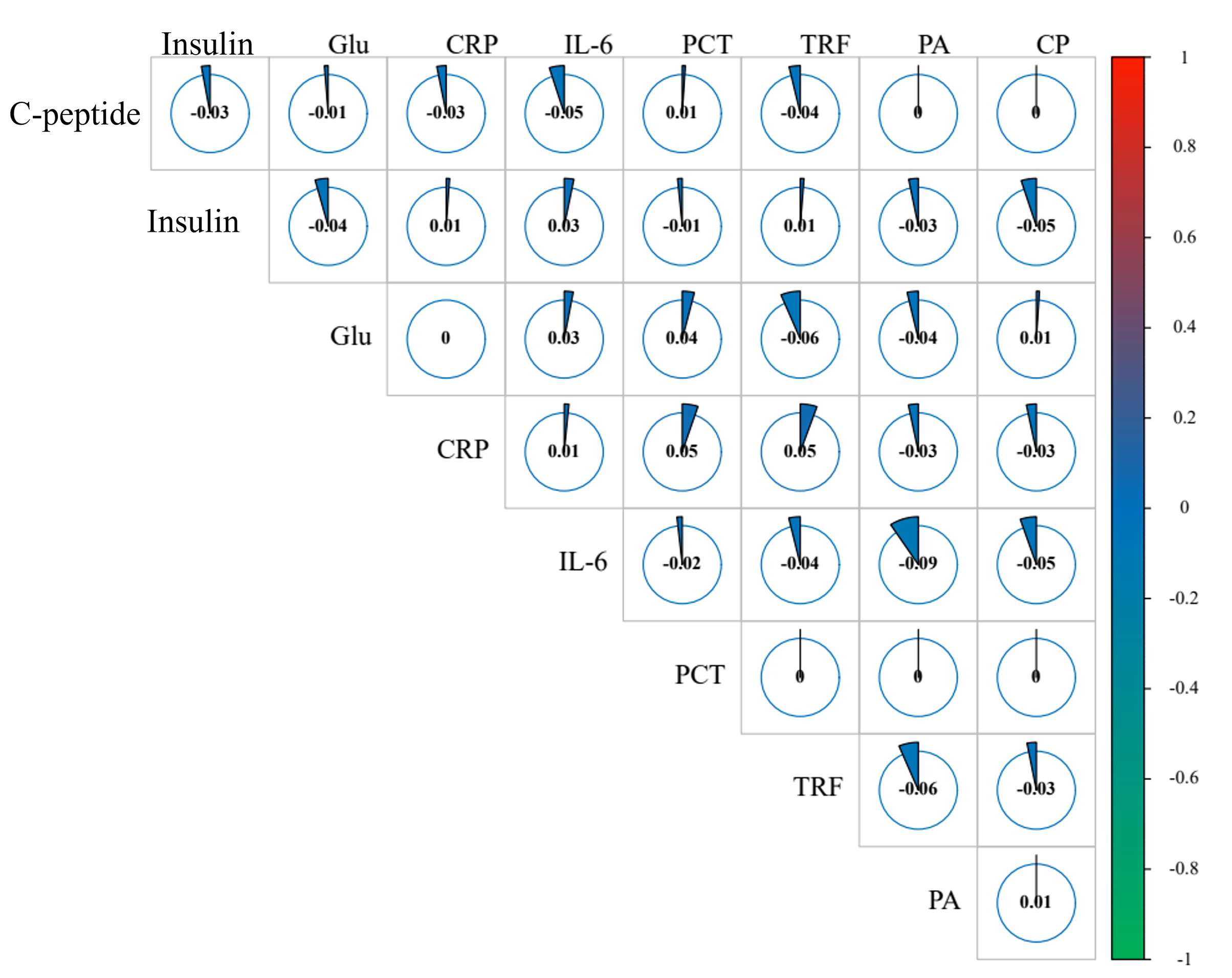

Supplement: Supplementary file 5 — FIGURE S5. Correlation analysis. Spearman correlation analysis was performed to analyze the relationship between blood glucose, insulin, C‐peptide, and the acute‐phase reactive proteins. The numbers in the figure represent the values of the correlation coefficients by spearman analysis. [file JCLA-39-e70052-s001.tif]
