# Supplementary material for: Fluctuations and Changes in Acute Phase Reactive Proteins in Fasting and Nonfasting States
Source: J Clin Lab Anal. 2025 May 10;39(12):e70052. doi: 10.1002/jcla.70052 (PMC12179803; doi:10.1002/jcla.70052)
Supplement: Supplementary file 6 — TABLE S1. Subgroup analyses of age in 50 subjects. [file JCLA-39-e70052-s006.docx]

**Table S1. Subgroup analyses of age in 50 subjects.**

| APRPs | Compared to T0 | **Age ≤ 50 year**  **(n=22)** | **Age >50 year**  **(n=28)** | **χ²** | ***P*** |
| --- | --- | --- | --- | --- | --- |
|  |  | Total number exceed Tea  n (%) | Total number exceed Tea  n (%) |  |  |
| CRP | T1 vs T0 | 5 (22.73%) | 12 (42.86%) | 1.900 | 0.593 |
|  | T2 vs T0 | 7 (31.82%) | 14 (50.00%) |  |  |
|  | T3 vs T0 | 9 (40.91%) | 14 (50.00%) |  |  |
|  | T4 vs T0 | 3 (13.64%) | 13 (46.43%) |  |  |
| IL-6 | T1 vs T0 | 12 (54.54%) | 18 (64.29%) | 0.473 | 0.925 |
|  | T2 vs T0 | 13 (59.09%) | 14 (50.00%) |  |  |
|  | T3 vs T0 | 12 (54.54%) | 16 (57.14%) |  |  |
|  | T4 vs T0 | 13 (59.09%) | 19 (67.86%) |  |  |
| PCT | T1 vs T0 | 7 (31.82%) | 7 (25.00%) | 0.677 | 0.879 |
|  | T2 vs T0 | 7 (31.82%) | 5 (17.86%) |  |  |
|  | T3 vs T0 | 6 (27.27%) | 8 (28.57%) |  |  |
|  | T4 vs T0 | 7 (31.82%) | 6 (21.43%) |  |  |
| TRF | T1 vs T0 | 7 (31.82%) | 11 (39.29%) | 0.869 | 0.833 |
|  | T2 vs T0 | 3 (13.63%) | 7 (25.00%) |  |  |
|  | T3 vs T0 | 5 (22.73%) | 8 (28.57%) |  |  |
|  | T4 vs T0 | 9 (40.91%) | 10 (35.71%) |  |  |
| PA | T1 vs T0 | 4 (18.18%) | 8 (28.57%) | 4.724 | 0.193 |
|  | T2 vs T0 | 8 (36.36%) | 3 (10.71%) |  |  |
|  | T3 vs T0 | 6 (27.27%) | 7 (25.00%) |  |  |
|  | T4 vs T0 | 6 (27.27%) | 11 (39.29%) |  |  |
| CER | T1 vs T0 | 2 (9.09%) | 8 (28.57%) | 0.646 | 0.886 |
|  | T2 vs T0 | 3 (13.64%) | 6 (21.43%) |  |  |
|  | T3 vs T0 | 2 (9.09%) | 6 (21.43%) |  |  |
|  | T4 vs T0 | 4 (18.18%) | 8 (28.57%) |  |  |
