# Supplementary material for: Fluctuations and Changes in Acute Phase Reactive Proteins in Fasting and Nonfasting States
Source: J Clin Lab Anal. 2025 May 10;39(12):e70052. doi: 10.1002/jcla.70052 (PMC12179803; doi:10.1002/jcla.70052)
Supplement: Supplementary file 7 — TABLE S2. Subgroup analyses for gender. [file JCLA-39-e70052-s008.docx]

**Table S2. Subgroup analyses for gender.**

| APRPs | Compared to T0 | **Male**  **(n=25)** | **Female**  **(n=25)** | **χ²** | ***P*** |
| --- | --- | --- | --- | --- | --- |
|  |  | Total number exceed Tea  n (%) | Total number exceed Tea  n (%) |  |  |
| CRP | T1 vs T0 | 13 (52.00%) | 4 (16.00%) | 0.206 | 0.977 |
|  | T2 vs T0 | 16 (64.00%) | 5 (20.00%) |  |  |
|  | T3 vs T0 | 17 (68.00%) | 6 (24.00%) |  |  |
|  | T4 vs T0 | 12 (48.00%) | 4 (16.00%) |  |  |
| IL-6 | T1 vs T0 | 15 (60.00%) | 15 (60.00%) | 0.504 | 0.918 |
|  | T2 vs T0 | 16 (64.00%) | 11 (44.00%) |  |  |
|  | T3 vs T0 | 15 (60.00%) | 13 (52.00%) |  |  |
|  | T4 vs T0 | 17 (68.00%) | 15 (60.00%) |  |  |
| PCT | T1 vs T0 | 8 (32.00%) | 6 (24.00%) | 0.345 | 0.951 |
|  | T2 vs T0 | 6 (24.00%) | 6 (24.00%) |  |  |
|  | T3 vs T0 | 8 (32.00%) | 6 (24.00%) |  |  |
|  | T4 vs T0 | 8 (32.00%) | 5 (20.00%) |  |  |
| TRF | T1 vs T0 | 8 (32.00%) | 10 (40.00%) | 1.647 | 0.649 |
|  | T2 vs T0 | 5 (20.00%) | 5 (20.00%) |  |  |
|  | T3 vs T0 | 8 (32.00%) | 5 (20.00%) |  |  |
|  | T4 vs T0 | 12 (48.00%) | 7 (28.00%) |  |  |
| PA | T1 vs T0 | 5 (20.00%) | 7 (28.00%) | 0.391 | 0.942 |
|  | T2 vs T0 | 5 (20.00%) | 6 (24.00%) |  |  |
|  | T3 vs T0 | 6 (24.00%) | 7 (28.00%) |  |  |
|  | T4 vs T0 | 9 (36.00%) | 8 (32.00%) |  |  |
| CER | T1 vs T0 | 7 (28.00%) | 3 (12.00%) | 0.234 | 0.972 |
|  | T2 vs T0 | 6 (24.00%) | 3 (12.00%) |  |  |
|  | T3 vs T0 | 6 (24.00%) | 2 (8.00%) |  |  |
|  | T4 vs T0 | 9 (36.00%) | 3 (12.00%) |  |  |
