# Supplementary material for: Fluctuations and Changes in Acute Phase Reactive Proteins in Fasting and Nonfasting States
Source: J Clin Lab Anal. 2025 May 10;39(12):e70052. doi: 10.1002/jcla.70052 (PMC12179803; doi:10.1002/jcla.70052)
Supplement: Supplementary file 8 — TABLE S3. Results of glucose interference experiments. [file JCLA-39-e70052-s003.docx]

**Table S3. Results of glucose interference experiments**

| PARPs | Value of PARPs (M ± SD) | | Mean bias (%) |
| --- | --- | --- | --- |
|  | 150μL serum+150uL FBS  (with 30mmol/L glucose) | 150μL serum  +150uL FBS |  |
| CRP (mg/L) | 1.67±0.01 | 1.71±0.05 | 2.39% |
|  | 9.31±0.18 | 8.90±0.23 | -4.60% |
|  | 20.93±0.75 | 20.70±1.71 | -1.11% |
| IL-6 (pg/mL) | 1.50±0.00 | 1.57±0.10 | 4.46% |
|  | 8.91±0.25 | 9.09±0.58 | 1.98% |
|  | 31.43±0.84 | 33.04±1.12 | 4.88% |
| PA (mg/L) | 54.60±0.85 | 54.47±0.61 | -0.24% |
|  | 130.00±9.80 | 131.33±13.30 | 1.01% |
|  | 196.67±14.38 | 191.33±9.67 | -2.79% |
| TRF (g/L) | 0.89±0.02 | 0.91±0.04 | 2.20% |
|  | 1.15±0.03 | 1.16±0.04 | 0.86% |
|  | 1.62±0.05 | 1.63±0.05 | 0.61% |
| PCT (mg/L) | 0.86±0.02 | 0.87±0.04 | 1.15% |
|  | 1.61±0.01 | 1.66±0.02 | 3.01% |
|  | 3.58±0.03 | 3.54±0.15 | -1.13% |
| CER (g/L) | 0.15±0.01 | 0.16±0.00 | 6.25% |
|  | 0.21±0.01 | 0.20±0.00 | -5.00% |
|  | 0.24±0.01 | 0.25±0.01 | -4.00% |

Each test was repeated 3 times and the data were described as mean ± standard deviation (M ± SD).
